# Home Anxiety Assessment and Influencing Factors among Adolescent Athletes in Yantai City

**DOI:** 10.3390/children11060628

**Published:** 2024-05-24

**Authors:** Yuxi Chen, Chunming Ye, Yang Lin, Yongjie Ma, Xingyu Zhang, Jiu Wang

**Affiliations:** 1 School of Public Health, Binzhou Medical University, Yantai 264003, China; yuxichen4608@163.com (Y.C.); 18463656434@163.com (Y.L.); tyf20230524@163.com (Y.M.); 2 Yantai Sports Industry Development Service Center, Yantai 264003, China; yechunming123@163.com; 3 School of Health and Rehabilitation Science, University of Pittsburgh, Pittsburgh, PA 15213, USA

**Keywords:** adolescent athletes, home anxiety, COVID-19 pandemic, influencing factors

## Abstract

Objective: To understand the prevalence of home-related anxiety among adolescent athletes during the novel coronavirus pandemic and to ascertain the factors influencing this anxiety. Methods: We employed cluster sampling to select 1150 adolescent athletes (aged 8–18 years) from six sports training schools in Yantai City, Shandong Province. Mental health status was assessed and recorded. Chi-square tests and multivariable logistic regression were used to analyze the factors contributing to athletes’ anxiety. Results: The survey revealed a COVID-19 infection rate of 38.23% (437 individuals) with an anxiety score of 40.98 ± 8.20 and an anxiety detection rate of 11.29% (129 individuals) during the COVID-19 epidemic. Female athletes exhibited a higher anxiety rate of 14.40% compared to 8.40% in male athletes. Multivariate analysis identified female gender as a risk factor for anxiety (OR = 1.64), while participation in aquatics emerged as a protective factor (OR = 0.24, 95% CI: 1.08–2.48). Professional training duration exceeding three years increased anxiety risk (OR = 3.05, 95% CI: 1.67–5.58), as did not seeking help during difficulties (OR = 2.59, 95% CI: 1.33–5.01). Interestingly, parental care was linked to increased anxiety risk (OR = 2.44, 95% CI 1.34–4.44), while care from friends was protective (OR = 0.60, 95% CI: 0.36–1.01), which was possibly due to the pressure associated with parental expectations. Conclusions: Adolescent athletes, particularly females and those with extended training durations, exhibit a heightened susceptibility to anxiety. This study also highlights that athletes who proactively seek assistance during challenging situations tend to experience lower anxiety levels. Additionally, a lack of COVID-19 infection and the involvement of concerned parents contribute to reduced anxiety among these young athletes.

## 1. Introduction

Throughout the evolving landscape of the COVID-19 pandemic since its initial outbreak in 2019, extensive global research has consistently illuminated the profound impact this ongoing crisis has had on the mental well-being of individuals. This body of research consistently reveals a surge in psychological symptoms, encompassing depression, anxiety, and stress, all of which harbor the potential for enduring consequences on individuals’ adaptive functioning [1,2,3]. As we find ourselves in the landscape of January 2023, it is undeniably clear that the shadow of the coronavirus pandemic continues to loom over China. This particular juncture, marked by the vacation period for young athletes, underscores the undeniable urgency of addressing their mental health [4]. Among these young athletes, a heightened vulnerability to anxiety is evident, accentuating the pressing need for timely well-being assessments and essential support to guide them through the unique challenges they presently confront [5,6]. During the COVID-19 pandemic, athletes have had to complete their learning and training at home, which has impacted their training efficiency and contributed to increased anxiety. An essential characteristic of an excellent athlete is the ability to effectively manage anxiety [7,8]. To improve their training status, athletes should dedicate time to learning about psychology and practicing techniques such as self-suggestion and self-motivation [9,10].

The cohort of young athletes faces a distinctive set of challenges during these trying times. They must navigate not only the rigorous pandemic containment measures but also the adaptation to a novel mode of education, involving remote learning from the comfort of their homes. Simultaneously, they remain resolute in their dedication to maintaining their physical fitness and excelling in their athletic pursuits [11,12]. Against the backdrop of the ongoing coronavirus epidemic, it has become imperative to focus on understanding and addressing the anxiety levels experienced by these young athletes [9]. Such dedicated attention holds pivotal significance in ensuring their holistic and healthy development [13,14]. The mental well-being of both elite and student-athletes has emerged as a pressing and vital subject, warranting a comprehensive examination and in-depth discussion within the current context [15,16,17].

This study represents a meticulous exploration of the levels of anxiety experienced by adolescent athletes within the confines of their homes during the tumultuous COVID-19 pandemic. It seeks to identify the key influencing factors through cluster sampling, which is a method that involved the selection of 1143 adolescent athletes aged between 8 and 18 years. These young athletes were drawn from six sports training schools located in the vibrant city of Yantai, China. Our study assesses the prevalence and determinants of anxiety among adolescent athletes in Yantai City during the COVID-19 pandemic. We aim to establish how widespread anxiety is among these athletes and explore factors like gender, age, sport type, and training duration that may influence their anxiety levels. Additionally, we examine the impact of various sources of social support on these athletes’ mental health, considering the isolating effects of the pandemic. We hypothesize that the prevalence of anxiety is significantly higher during the pandemic compared to pre-pandemic levels. We also predict that female athletes and those with longer training durations experience greater anxiety with individual sports participants showing higher anxiety levels than team sports participants due to less team-based support. Furthermore, we expect that athletes receiving more social support from non-familial sources, such as coaches and peers, will report lower anxiety levels. Lastly, we suggest that athletes who proactively seek help during challenging times will have lower anxiety levels than those who do not.

## 2. Data and Methods

The schools selected for participation in our study include the Yantai Sports School, Yantai Water Sports School, Yantai Cycling School, Yantai Shooting and Archery Center, Longkou Sports School, and Zhifu Sports School. These institutions, all located in Yantai City, Shandong Province, were chosen based on their accessibility and willingness to participate, which facilitated the logistical aspects of conducting our research. Our target demographic consisted of adolescent athletes aged between 8 and 18 years. The data collection period spanned from 10 January to 20 January 2023, during which we utilized the “sojump” network survey platform for an online survey. We distributed a total of 1150 questionnaires and successfully collected 1143 valid questionnaires, resulting in an impressive effective rate of 99.39%.

The General Information Questionnaire was employed to gather demographic information about the participants, such as gender, age, sibling status, and home address. The COVID-19 Infection Questionnaire was utilized to ascertain the COVID-19 infection status of the study participants, including their infection history, vaccination status, and types of COVID-19 infection. The Social Support Information Survey, based on the Perceived Social Support Scale (PSSS), was administered to assess the social support status of young athletes (Supplementary Table S1) [18,19]. To gauge the subjective experience of anxiety symptoms among our subjects, we employed the Self-Rating Anxiety Scale (SAS) (Supplementary Table S2). This scale comprises 20 items, each with four possible ratings: A (no or very little time), B (sometimes), C (most of the time), and D (most or all of the time). For scoring purposes, we assigned values to these ratings as follows: A = 4 points, B = 3 points, C = 2 points, and D = 1 point for items 5, 9, 13, 17, and 19; for the remaining questions, A = 1, B = 2, C = 3, D = 4. The overall score was calculated by summing the scores of all 20 questions, and the raw score was then multiplied by 1.25 to yield the standard score. Building on the approach used in the previous study [20,21], we classified anxiety rating scores as follows: scores below 49 were categorized as “not anxious,” and scores of 49 and above were categorized as “anxious.” Within this, scores from 50 to 59 denoted mild anxiety, scores from 60 to 69 indicated moderate anxiety, and scores exceeding 69 were considered indicative of severe anxiety.

We conducted an analysis using SPSS software to evaluate the reliability and validity of the instruments used in our study. Our findings confirm the high reliability and validity of the PSSS, as evidenced by an impressive internal consistency reliability score of 0.93. Such a high score indicates that the items within the PSSS are consistently measuring the same underlying concept of social support, making it a reliable tool for our research purposes. Furthermore, the SAS demonstrated excellent measurement properties. It achieved a Cronbach’s alpha coefficient of 0.841, indicating strong internal consistency among the items and suggesting that they effectively capture varying levels of anxiety. The Kaiser–Meyer–Olkin (KMO) measure of sampling adequacy returned a value of 0.891, which far exceeds the acceptable threshold of 0.5 and underscores the appropriateness of the factor analysis conducted on our dataset. Additionally, the Bartlett’s test of sphericity was significant at *p* < 0.01, confirming that the variables are sufficiently correlated for factor analysis, hence justifying the structure of the scale. These statistical indicators collectively validate the reliability and validity of our measurement tools. The high scores and significant test results ensure that our measures adhere to the necessary standards for conducting sound, reliable statistical analyses in psychological research. This robust validation empowers us to draw well-supported conclusions from our data, contributing to the reliability of our overall findings and enhancing the credibility of our study within the scientific community.

We uniformly distributed the electronic questionnaire to the participating schools. Prior to distributing the questionnaire, we provided standardized instructions to the subjects, emphasizing the importance of anonymity to ensure the integrity and accuracy of the responses. The electronic questionnaire was disseminated uniformly across the selected schools. Before distribution, we employed uniform guidance to instruct the subjects on how to complete the questionnaire, emphasizing the importance of careful and accurate completion to obtain reliable results.

### 2.1. Quality Control

In the initial design phase of our study, meticulous attention was dedicated to crafting a comprehensive questionnaire. We derived valuable insights from an extensive literature review and pertinent policies to inform the questionnaire’s content. To enhance the authenticity of respondents’ answers, the questionnaire was thoughtfully structured, featuring clear explanations and detailed instructions for addressing key issues. Furthermore, we actively sought feedback from experts specializing in kinesiology and psychology. Their valuable input was instrumental in refining the questionnaire, thereby ensuring its validity and relevance.

During the investigation stage, as we administered the questionnaire, our approach emphasized clarity and participant understanding. Detailed explanations were provided to participants, and conducive conditions were set to facilitate questionnaire completion. Our project team adopted a systematic approach with tasks divided among team members to encourage a thorough and attentive response from participants. This approach effectively minimized the likelihood of omissions and prevented duplicate submissions.

Upon reaching the Data Analysis Stage, data were extracted directly from the questionnaire platform. To maintain data integrity, two dedicated data managers conducted meticulous checks to identify and rectify any logical errors. Following this data quality assurance process, the collected data were meticulously organized, and a comprehensive logical verification was conducted to identify and address any data inconsistencies. Subsequently, we employed appropriate statistical analysis methods to derive meaningful insights and draw conclusions from the dataset. This methodical approach ensured the reliability and accuracy of our data analysis, contributing to the overall rigor of our study.

### 2.2. Statistical Analysis

A descriptive analysis was conducted to elucidate the differences in anxiety detection rates across various demographic characteristics. Chi-square tests were used for univariate analysis to examine the detection rate of anxiety in adolescent athletes under different COVID-19 infection situations and social support scenarios. Logistic regression analysis was undertaken with the presence or absence of anxiety being considered as the dependent variable. The multivariable analysis employed a backward variable selection procedure with a 0.2 entry criteria. The goal was to identify the influencing factors responsible for the occurrence of anxiety. The significance level (α) was set at 0.05, and the data analysis was carried out using SPSS (Statistical Package for the Social Sciences) Version 26.0.

We calculated our sample size and power based on the objective to identify factors influencing the occurrence of anxiety. Our study collected data from 1143 participants, approximately 50% of whom exhibited symptoms of anxiety. Using univariate logistic regression with a significance level of 0.05 and a power of 0.80, we are able to detect an effect size corresponding to a standardized odds ratio of 0.84 or 1.18, which is considered a small effect size [22].

## 3. Results

In the study, a total of 1143 participants were included (Table 1). Among them, 595 were boys (52.06%), and 548 were girls (47.94%). The distribution between urban and rural areas was fairly balanced with 599 (52.41%) residing in urban areas and 544 (47.59%) in rural areas. Participants had varying durations of special training, with 338 (29.57%) having 1 year of training, 331 (28.96%) having 1 to 2 years, 193 (16.89%) having 2 to 3 years, and 281 (24.58%) having 3 to 3 years of training. Regarding sports involvement, 123 (10.76%) participated in track and field events, 185 (16.19%) in water events, 266 (23.27%) in ball events, 210 (18.37%) in shooting events, 275 (24.06%) in heavy sports events, and 84 (7.35%) in other sports events.

The age range of the participants was 8 to 18 years old with an average age of 13.78 (±1.90). The results of the study showed that out of the total sample, 129 individuals exhibited symptoms of anxiety, resulting in an anxiety rate of 11.29%. In relative terms, the prevalence of anxiety among adolescent athletes was in the minority. The anxiety detection rate was higher among females (14.4%) compared to males (8.4%), and this difference was statistically significant (*p* = 0.0013). Athletes in the older age group (15.0%) had a higher anxiety detection rate compared to those in the middle age group (9.7%) and the younger age group (5.9%) (*p* = 0.0139). Water sports athletes had the lowest anxiety detection rate (2.7%), which was significantly lower than track and field athletes (15.5%), weight lifting athletes (17.1%), and shooting athletes (17.1%) (*p* = 0.0001). Athletes with over three years of professional training (19.9%) had a substantially higher anxiety detection rate than those with less than one year of training (5.3%) (*p* = 0.001). The level of support from parents, coaches, friends, and family was statistically significant (*p* < 0.01). Athletes who frequently sought help when facing difficulties (7.0%) had a lower anxiety detection rate than those who never sought help and those who occasionally sought help (17.1% and 25.9%), and this difference was statistically significant (*p* = 0.0007).

**Figure 1:**
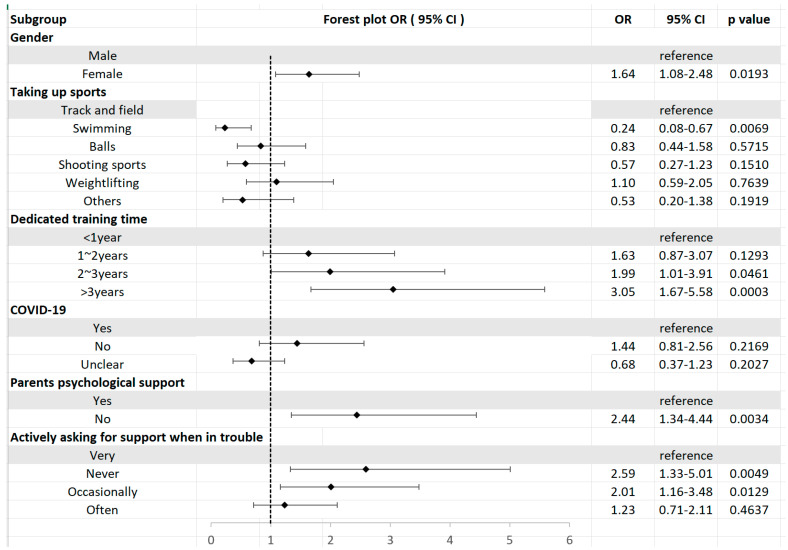
Forest plot of odds ratios for anxiety.

In the multivariable analysis (Table 2 and Figure 1), several factors were identified as significant contributors to anxiety. Gender, involvement in sports, duration of professional training, parental concern, and seeking help during difficulties emerged as key influencers of anxiety levels. Gender played a significant role with females being 1.64 times more likely to experience anxiety compared to males (95% CI: 1.08–2.48). Athletes involved in aquatics demonstrated a 24% lower likelihood of experiencing anxiety in comparison to their counterparts in track and field events (OR: 0.24, 95% CI: 1.08–2.48). Athletes with over three years of professional training exhibited 3.05 times higher odds of anxiety when contrasted with those who had less than one year of training (95% CI: 1.67–5.58). Athletes who received care and support from their parents were 2.44 times more likely to experience anxiety than those who did not receive such support (OR: 2.44, 95% CI: 1.34–4.44). Athletes who never sought assistance when confronted with difficulties had a 2.59 times higher likelihood of anxiety in comparison to those who frequently sought help. Conversely, athletes who never sought help when facing challenges had a 2.59 (95% CI: 1.33–5.01) times higher chance of experiencing anxiety than those who consistently sought assistance.

## 4. Discussion

Our study identifies a significant elevation in anxiety levels among adolescent athletes in Yantai City during the COVID-19 pandemic, highlighting a critical area of concern in sports psychology and youth athletic programs. Particularly, female athletes and those undergoing extensive training durations exhibited higher anxiety levels, suggesting that gender-specific factors and the pressure of intense training schedules contribute significantly to mental health risks. Additionally, athletes participating in individual sports reported higher anxiety compared to those in team sports, emphasizing the social support component inherent in team environments that may buffer against psychological stress. The role of non-familial social support, especially from coaches and peers, emerged as a crucial protective factor against anxiety. This finding points to the potential of these relationships to offer emotional support that is less fraught with the expectations and complexities often associated with familial interactions. Athletes reporting strong support networks were notably less anxious, indicating that enhancing communication channels and support mechanisms within sports teams could be particularly beneficial. Moreover, athletes who proactively sought help when facing difficulties demonstrated lower levels of anxiety, underscoring the importance of encouraging help-seeking behavior as a coping strategy. This proactive approach not only aids in immediate stress reduction but also builds resilience over time, preparing athletes to handle future challenges more effectively.

The outbreak of novel coronavirus pneumonia occurred during the winter season, coinciding with the peak of influenza. However, the early stages of novel coronavirus pneumonia exhibited symptoms similar to the common flu, leading to potential misdiagnosis. At that time, effective antibacterial drugs for the epidemic pathogen were lacking. As a result, even minor illnesses among athletes could trigger self-doubt, increase psychological pressure, and induce anxiety [7,23].

In addition to their rigorous physical training, young athletes are also required to excel in their academic studies. The pandemic disrupted students’ learning routines to varying degrees. Studies have shown that Chinese college students’ adaptability to online learning was influenced by several factors, including their online learning experience, network and electronic equipment accessibility, home learning environment, learning platform, and available learning resources [8,24]. Inadequate learning environments could lead to decreased learning capabilities, affecting academic performance. This, in turn, impacts the efficiency of sports training, potentially leading to mental health issues such as anxiety, depression, and irritability [25,26,27,28].

Adolescent athletes, still in the midst of their developmental stage, often lack adequate coping mechanisms for psychological challenges. Facing intense and high-stakes competitions, athletes naturally experience nervousness and unease [29,30,31]. However, high-quality athletes possess the ability to proactively manage anxiety and stress through various coping strategies. Research indicates that elite athletes, especially those with advanced skill levels, employ flexible and diverse methods to tackle tension. They employ techniques such as self-suggestion, situational imitation, relaxation techniques, and psychological guidance to mitigate the impact of anxiety during sports competitions, enabling them to perform at their best [32,33,34]. Thus, effective stress management is considered a crucial positive psychological trait for accomplished athletes [35].

To nurture the qualities that foster psychological resilience and alleviate state anxiety among adolescent athletes, a concerted effort involving multiple stakeholders is paramount in order to develop collaborative measures that can be taken by each group.

Young athletes should proactively work toward maintaining balanced schedules that accommodate their academic commitments, physical training, and the imperative need for adequate rest. They should be actively engaged in seeking effective solutions to mitigate the adverse impacts of anxiety on their performance and overall well-being. Prioritizing physical fitness and health, along with gaining a comprehensive understanding of the novel coronavirus and adhering to safety protocols, is of utmost importance. In moments of adversity, reaching out for support and guidance from coaches and teammates is strongly encouraged. Moreover, athletes should place a strong emphasis on personal development by enhancing their psychological resilience. This involves meticulous preparation for competitions, agile adjustments of their mental state during events, unwavering concentration, and thorough self-assessment post-game. Additionally, they should invest time in acquiring psychological knowledge and practicing techniques such as self-suggestion and self-motivation to refine their competitive mindset. For female athletes, heightened awareness and proactive communication regarding concerns and challenges are vital steps toward reducing anxiety [9,10].

Coaches hold a pivotal role in nurturing athletes’ mental well-being. It is imperative for coaches to maintain a balanced perspective on competition results, refraining from imposing excessive demands that could inadvertently create undue pressure. Tailoring personalized approaches to provide home learning mentality guidance is key. These tailored approaches should be crafted based on individual athletes’ needs and considerations of influencing factors, thereby contributing to the athletes’ mental health and overall development.

Parents are integral in providing unwavering support to their children. While offering assistance, parents should be mindful to avoid excessive concern, which may inadvertently intensify the pressure experienced by their children, potentially exacerbating anxiety. Parents should serve as a guiding light for athletes, helping them view competition results from a rational standpoint. Adolescents often harbor irrational beliefs about competition, and parents can play a crucial role in assisting them in developing a more realistic perspective through open and effective communication.

Relevant authorities and organizations have a crucial role to play in shaping the mental resilience of young athletes. They should actively organize a diverse array of youth competitions to provide ample opportunities for young athletes to engage in competitive events. Such competitions serve as valuable training grounds, enabling athletes to build psychological resilience when confronted with intense competition scenarios. Encouraging coaches and athletes to participate in seminars and knowledge-sharing sessions can foster the exchange of valuable experiences. This exchange, in turn, contributes to the overall mental health and development of the youth sports system.

Limitations of the study are acknowledged, including a wide age range of participants (8–18 years), which may affect the generalizability of the findings. The use of self-report questionnaires could introduce response bias. The cross-sectional design limits our ability to establish causality between identified factors and anxiety levels. Additionally, the study was conducted in a specific geographic region, which may limit the generalizability of the findings to other populations. Future research should address these limitations by employing a more diverse sample, longitudinal designs, and considering a broader range of influencing factors to provide a more comprehensive understanding of anxiety among adolescent athletes.

## 5. Conclusions

Our research highlights a substantial rise in anxiety levels among adolescent athletes in Yantai City during the COVID-19 pandemic. The increase was particularly pronounced among female athletes and those engaged in extensive training periods, indicating these groups are at greater risk. The findings also emphasize the beneficial effects of team sports and support from non-family members, such as coaches and peers, in reducing anxiety levels. Based on these observations, there is a clear need to incorporate psychological support and proactive coping mechanisms within athletic training programs to enhance mental well-being. Additionally, future research should investigate the long-term impacts of pandemic-induced anxiety and assess the effectiveness of specific interventions designed to support the mental health of athletes. The ultimate goal of our study is to offer actionable insights that aid in the development of effective prevention strategies against pandemics and tailored psychological support for the specific challenges faced by young athletes.

## Figures and Tables

**Table 1:** Anxiety detection rates stratified by subgroups among athletes.

Subgroup	Overall Count	Count of Anxiety	Anxiety Proportion %	*p* Value
	1143	595	52.06	
Gender				
Male	595	50	8.4	0.0013
Female	548	79	14.42	
Age, years				
8–10	51	3	5.88	0.0139
11–14	704	68	9.66	
15–19	388	58	14.95	
Home residence type				
Urban	599	69	11.52	0.7939
Rural	544	60	11.03	
Sports type				
Track and field	123	19	15.45	0.0001
Swimming	185	5	2.7	
Balls	266	36	13.53	
Shooting sports	210	15	17.14	
Weightlifting	275	47	17.09	
Others	84	7	8.33	
Professional training length				
<1 year	338	18	5.33	0.0001
1~2 years	331	32	9.67	
2~3 years	193	23	11.92	
>3 years	281	56	19.93	
COVID-19				
Yes	437	69	15.79	0.0001
No	532	40	7.52	
Unclear	174	20	11.49	
Parents psychological support				
Yes	1047	108	10.32	0.0006
No	96	21	21.88	
Coach care psychological support				
Yes	416	34	8.17	0.0119
No	727	95	13.07	
Teammates psychological support				
Yes	143	18	12.59	0.299
No	1000	111	11.1	
Friends psychological support				
Yes	139	28	20.1	0.0001
No	1004	101	10.1	
Grandparents psychological support				
Yes	172	20	11.6	0.8779
No	971	109	11.2	
Psychological support from other relatives				
Yes	31	4	12.9	0.773
No	1112	125	11.2	
Frequency of family support				
Never	6	1	16.67	0.0007
Occasionally	59	17	28.81	
Often	292	32	10.96	
Very	786	79	10.05	
Actively asking for support when in trouble				
Never	123	21	17.1	0.0007
Occasionally	271	43	25.9	
Often	392	40	10.2	
Very	357	25	7	

**Table 2:** Logistic regression analysis of factors influencing anxiety.

	Crude OR			Adjusted OR		
	OR	95% CI	*p* Value	OR	95% CI	*p* Value
Gender						
male	reference			reference		
female	1.84	1.26–2.67	0.0015	1.64	1.08–2.48	0.0193
Age, years						
8–10	reference			reference		
11–14	1.71	0.52–5.64	0.3778			
15–19	2.81	0.85–9.33	0.0911			
Home residence type						
Urban	reference			reference		
Rural	0.95	0.66–1.38	0.7938			
Sports type						
Track and field	reference			reference		
Swimming	0.15	0.06–0.42	0.0003	0.24	0.08–0.67	0.0069
Balls	0.86	0.47–1.56	0.6148	0.83	0.44–1.58	0.5715
Shooting sports	0.42	0.21–0.86	0.0181	0.57	0.27–1.23	0.1510
Weightlifting	1.13	0.63–2.02	0.6838	1.10	0.59–2.05	0.7639
Others	0.50	0.20–1.24	0.135	0.53	0.20–1.38	0.1919
Professional training length						
<1 year	reference			reference		
1~2 year	1.90	1.05–3.65	0.0352	1.63	0.87–3.07	0.1293
2~3 years	2.41	1.26–4.58	0.0076	1.99	1.01–3.91	0.0461
>3 years	4.43	2.53–7.73	0.0001	3.05	1.67–5.58	0.0003
COVID-19						
No	reference			reference		
Yes	1.44	0.85–2.46	0.1761	1.44	0.81–2.56	0.2169
Unclear	0.63	0.36–1.10	0.1051	0.68	0.37–1.23	0.2027
Parents psychological support						
No	reference			reference		
Yes	2.43	1.44–4.11	0.0009	2.44	1.34–4.44	0.0034
Coach care psychological support						
No	reference			reference		
Yes	1.69	1.12–2.55	0.0126			
Teammates psychological support						
No	reference			reference		
Yes	0.87	0.51–1.48	0.5993			
Friends psychological support						
No	reference			reference		
Yes	0.44	0.28–0.70	0.0006	0.60	0.36–1.01	0.0542
Grandparents psychological support						
No	reference			reference		
Yes	0.96	0.58–1.60	0.8778			
Psychological support from other relatives						
No	reference			reference		
Yes	0.85	0.29–2.48	0.7732			
Frequency of family support						
Very	reference			reference		
Never	2.02	0.22–18.63	0.5336			
Occasionally	0.62	0.07–5.43	0.6622			
Often	0.56	0.06–4.84	0.5973			
Actively asking for support when in trouble						
Very	reference			reference		
Never	2.73	1.47–5.09	0.0015	2.59	1.33–5.01	0.0049
Occasionally	2.51	1.49–4.22	0.0006	2.01	1.16–3.48	0.0129
Often	1.51	0.90–2.54	0.1221	1.23	0.71–2.11	0.4637

Note: The multivariable analysis employed a backward variable selection procedure with a 0.2 entry criteria.

## Data Availability

The data that support the findings of this study are available on request from the corresponding author upon reasonable request. The data of this study cannot be publicly obtained due to the reason of involving the survey objects.

## Supplementary Materials

The following supporting information can be downloaded at: https://www.mdpi.com/article/10.3390/children11060628/s1, Table S1: Selected perceived social support scale questions used in the study. Table S2: self-rating anxiety scale SAS.
