# Supplementary material for: Home Anxiety Assessment and Influencing Factors among Adolescent Athletes in Yantai City
Source: Children (Basel). 2024 May 24;11(6):628. doi: 10.3390/children11060628 (PMC11201604; doi:10.3390/children11060628)
Supplement: Supplementary file 1 [file children-11-00628-s001.zip › children-2964075-supplementary.pdf]

Supplement Table S1. Selected Perceived Social Support Scale Questions Used in the Study.

A Way to Ask for Help When You Are in the Most Pain and Suffering?	On one's own	Rarely asks for help	Sometimes ask others for help	Always ask for help	
Do Your Family Care About You When Things Get Tough?	Never care	Show little concern	Always care	Are very concerned	
Who is Taking Care of You After COVID-19?	Parent	Grandparents	Relative	Oneself	Other
Currently, You Are Not Infected with the New Coronavirus. When You Are Worried About Being Infected, Who Can Support You?	Parent	Grandparents	Relative	Oneself	Other

Supplement Table S2. Self-Rating Anxiety Scale SAS.

There are 20 questions below. Please read each one carefully. After each text, there are four choices: A has none or very little, B has some, C has most or all the time, and D has most or all of the time. According to the actual situation of the past two weeks, choose the appropriate option.

	A	B	C	D
1. I feel more nervous and anxious than usual.				
2. I feel scared for no reason.				
3. I am easily upset or frightened.				
4. I think I might go mad.				
5. I think everything is fine and nothing bad will happen.				
6. My hands and feet are trembling; my hands and feet are quite shaky.				
7. I suffer from headaches, neck pain, and back pain.				
8. I feel weak and tired easily.				
9. I feel calm and find it easy to sit still.				
10. I feel my heart beating fast.				
11. I suffer from bouts of dizziness.				
12. I have a fainting attack or feel like I'm going to faint.				
13. I find it easy to breathe in and out.				
14. I have numbness and tingling in my hands and feet.				
15. I suffer from stomachaches and indigestion.				
16. I often have to urinate.				
17. My hands are often dry and warm.				
18. My face is red and hot.				
19. I fall asleep easily and sleep well all night.				
20. I had nightmares.				
